# Standardization of B19 IgG ELISA to study the seroepidemiology of parvovirus B19 in North Indian voluntary blood donors

**DOI:** 10.4103/0973-6247.67022

**Published:** 2010-07

**Authors:** Janak Kishore, Manisha Srivastava, Nabjyoti Choudhary

**Affiliations:** Department of Microbiology, Sanjay Gandhi Post Graduate Institue of Medical Sciences, Lucknow-226014, India; 1 Assistant Professor, Bhopal Memorial Hospital & Research Center, Bhopal, MP, India; 2 Senior Consultant, Tata Medical Center, Rajarhat, Kolkata-700136, India

**Keywords:** Antibodies, blood donors, ELISA, erythrovirus, parvovirus B19, seroprevalence

## Abstract

**Backgound and Objectives:**

Parvovirus B19 (B19) being a non-enveloped DNA virus is hence thermo-stable to the current methods of viral inactivation. Therefore transfusion of blood or its component from a viremic donor to non-immune recipients may result in transfusion-transmitted B19 infection with occasional sinister complications. The serologically naïve blood donor population in our country has not been studied. Hence a study was designed to find the sero-status of B19 virus in normal voluntary blood donor population (relatives of recipients) as an indirect measure of the susceptibility to B19 in north Indians together with seroepidemiology of B19.

**Methods:**

An in-house anti-B19 IgG ELISA was standardized using cloned, baculovirus expressed, and purified VP1/VP2 capsid proteins as antigen. Anti-B19 IgG antibodies in sera (diluted 1:400) of 1000 healthy voluntary blood donors (18-60 years; mean 30.5 years) were analyzed and their epidemiologic data were documented.

**Results:**

A total of 399 (39.9%) donors were seropositive for B19 virus. Seroprevalence was higher in males than females (44% vs 27%) and it increased with increasing age (*P*<0.01). Socioeconomically, B19 IgG antibody positivities were 61.8%, 61.1%, and 44.4% in low, medium, and high income groups respectively with unskilled laborers having higher seroprevalence in low (48.5%) and middle (58.7%) income group (*P*<0.05). Housing conditions revealed B19 seroprevalence as 42.6% in donors living in small houses compared to 20.4% in larger houses (*P*<0.01) but no difference with religion.

**Conclusions:**

Seroprevalence to B19 in normal voluntary blood donors was low leaving a large proportion of north Indians susceptible to B19 infection.

## Introduction

Parvovirus B19 (B19) is the smallest, non-enveloped single-stranded DNA virus belonging to the family Parvoviridae in the recently created genus Erythrovirus.[1] B19 virus has a broad spectrum of clinical manifestions.[2,3] The virus was discovered by Yvonne Cossart in 1974 during screening of healthy blood donors for hepatitis B virus.[4] Transmission of B19 infection in a susceptible host occurs through transfusion of blood and blood components besides aerosol (droplets) and transplacental routes.[5–7] The B19 virus is present worldwide and seroepidemiological studies have shown that 40-60% of the world’s adult population have antibodies specific for B19 and immunity to B19 infection depending upon previous exposure and the presence of neutralizing anti-B19 IgG antibodies, mainly directed to VP1 capsid proteins of the virus.[3] Although most of the infections caused by B19 remains asymptomatic are self-limiting it can cause significant morbidity and occasional persistent infections or mortality in humans as also observed by us.[8–10]

It is still not known whether screening for parvovirus B19 IgG antibodies should be introduced for routine blood donors.[11] There have been reports from various countries in the world regarding the seroprevalence of B19 infection ranging from as low as 16.2% in Singapore, 32.8% to as high 80% in Japan, 75% in Belgium, 60% in England, 49% in America, 40-46.8% in Germany, and 64% in North Africa.[12–16] However no large series seroepidemiological studies in adults or donor population are available from India except limited data in children.[17,18] The present study was designed to find the seroepidemiology of B19 in healthy voluntary blood donor’s population from a developing country like India. As per policies of our institute, voluntary blood donors are usually relatives or familial personnel of patients mostly from far flung areas of north India hence they may be treated as indirect representative of general population of this region. Further in most of the reports B19 antigen used has been plasma derived, while in our study we have used cloned baculovirus expressed and purified VP1 and VP2 capsid proteins as antigen in ELISA test standardized in house.

## Materials and Methods

### Subjects

A total of 1000 healthy voluntary blood donors’ population attending the blood center of the department of Transfusion Medicine of Sanjay Gandhi Post-Graduate Institute of Medical Sciences, Lucknow (India) were taken randomly as subjects for this study. Serum samples from the voluntary blood donors were drawn for routine testing as recommended for blood banks but an aliquot was labeled and preserved at –70 °C for anti-B19 IgG antibodies by an in-house ELISA which was standardized and tested in the department of Microbiology of the institute. At the time of personal interview, the donors were subjected to a set of questionnaire that included relevant information regarding parvovirus infection.

### Indirect in-house ELISA(Qualitative):

**Figure 1:**
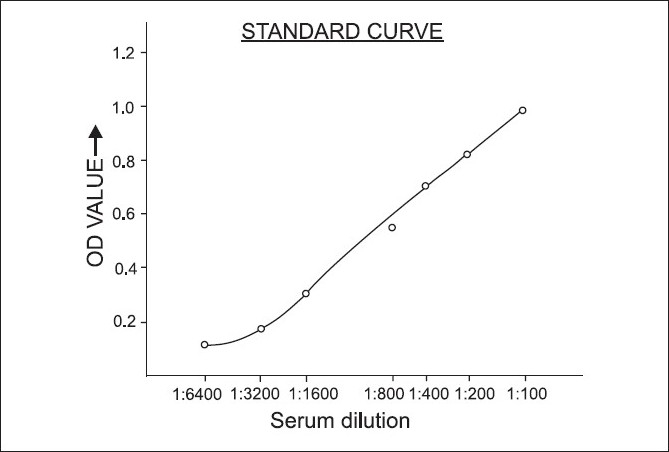
The standard graph showing near sigmoid line indicating specificity of immunological binding between antigen and antibodies of parvovirus B19

Anti-B19 IgG antibodies were detected by in-house indirect ELISA using cloned and baculovirus expressed and purified B19 empty capsid proteins as antigens (kindly donated by Dr Y. Matsunaga of NIH, Nagoya, Japan) and which immunologically react as similar to natural viral protein antigens.[19] The purified VP1 and VP2 proteins were mixed in equal proportions and used as antigen in the ELISA test. Firstly in-house indirect ELISA was standardized by finding optimum in use dilutions of cloned antigens and conjugate (rabbit-anti human IgG-HRP; Dako, Denmark) by the checker-board titrations method using a known positive sera. Subsequently, optimum dilutions of in use patient’s sera were determined by testing a set of known positive and negative sera (kindly donated by Dr Y. Matsunaga of NIH, Nagoya, Japan) which were diluted 1:50 to 1:6400 to find the specificity of antigen and antibody binding [Figure 1] and then tested on a series of B19 infected and PCR confirmed sera for cross reactivities specially with viruses causing rash and similar clinical manifestations such as rubella, herpes simplex virus varicella zoster, and Epstein Barr virus. The dilution of serum samples at 1: 400 gave the best discrimination between positive and negative controls. Hence, sera were diluted to 400, and antigen and conjugate to 5000 in phosphate buffer saline with 0.5% Tween-20 (PBS-T). Cloned, baculovirus expressed and purified VP1 and VP2 capsid proteins of B19 mixed in equal quantities were diluted 1: 5000 in carbonate buffer (Sigma, C-3041), and 100 μl (or 0.5 μg protein/well) of this solution was dispensed in each well of 96-well ELISA plate (NUNC, Maxisorb, USA). For coating of antigen, the plates were incubated for 37°C for 1 h and then at 4°C for overnight. On the second day, the plates were washed three times with PBS-T, 300 μl/well each time for 5 min, then blocking was done with 5% fetal calf serum (Gibco, BRL, USA) in PBS-T (200μl/well at 37°C for 1 h). Test sera and set of positive and negative control sera were diluted 1:400 and each sample was dispensed in three wells (100 μl/well at 37°C for 1 h). Anti-human IgG conjugate (Sigma, A-8650) was used at a dilution of 1:5000 in PBST (100 μl/well at 37°C for 1 h). Three washing by PBST followed every step. For color development tetramethyl benzidine (Banglore Geni, SFE-1, India) 100 μl/well was used. Reaction was stopped by adding 1M H_2_SO_4_, 50 μl/well, and O.D. (optical density) was taken at 450 nm in the ELISA reader (Biotech, USA). The cut-off O.D. value was determined by calculating mean ± 2SD of O.D. values of 1000 sera analyzed. The mean came out to be 0.3214 and SD was 0.1396; hence, the cutoff value decided was 0.600 OD at 450 nm. Final O.D. values for each sample were taken as the mean O.D. of three wells used per sample. If there was higher variation in values of three wells of same sample, the values were discarded and sample was tested again.

### Statistical methods:

A computerized analysis of data (OD) values with various parameters were carried out and the software program used were MSTAT and SPSS. The chi-square test was used to evaluate possible association between IgG positivity and other variables used in this study. Using chi-square and Fisher’s exact and Z tests calculated the probability value of differences between the positivity in different study groups.

## Results

Immunological binding between antigen and anti-B19 IgG antibodies was found to be specific as the standard graph [Figure 1] yielded a near sigmoid curve in the indirect in-house ELISA (qualitative) test and no cross-reactions were observed with sera taken from cases with viruses causing rash and similar clinical manifestations such as rubella, herpes simplex virus, varicella zoster, and Epstein Barr viruses, while it reacted specifically with serial sera taken from B19 infected cases and who were either anti-B19 IgM positive and anti-B19 IgG negative but seroconverted later on follow up.

After finding the validity of our anti-B19 IgG ELISA test, we analyzed sera taken from 1000 voluntary blood donor taken randomly and found overall seroprevalence of 39.9% (399 out of 1000). The mean age of the donors was 30.54 ± 7 years and ranged from 18 to 60 years. Table 1 shows a seroprevalence of 40.3%, 37.1%, 44.5%, and 50% in the age groups 18-30, 30-40, 40-50, 50-60 years, respectively. This observation suggests that as the age advances the prevalence of parvovirus B19 infection increase.

**Table 1:** Age distribution in relation to anti B19 IgG antibodies in north Indian blood donors

Age group (years)	Total no. of donors	Positive for B 19 IgG(%)	Negative for B 19 IgG(%)
18-30	493	199(40.3)	294(59.6)
30-40	350	130(37.1)	220(62.8)
30-40	137	61(44.5)	76(55.5)
50-60	20	10(50)	10(50)

Significantly higher prevalence was in older age group (50-60 years) in comparison to both younger age groups (18-30 years and 30-40 years) (*P*<0.01)

A total of 945 (94.5%) donors were men and only 55(5.5%) were women. The male preponderance was also observed in terms of positivity for anti-B19 IgG antibodies. As many as 384(40.6%) males and 15(27.3%) females were positive for anti-B19 IgG antibodies (*P*<0.01). The prevalence was 40.2% (191/474) in the males of age group 18-30 years, 38.5% in the age group 30-40 years (125/325), 46.03% (58/127) in age group 40-50 years, and 52% (10/19) in the age group 50-60 years. Thus it was noted that as the age increased, the prevalence increased in males but the number of females in each group was too small to draw any definite conclusions.

Marital status did not affect the prevalence in the study group. No statistically significant difference (*P*<0.05) in the positivity was evident among the married donors (39.5%) and the unmarried donors (40.8%). Maximum donors (92.4%) were followers of Hindu religion in this study and the prevalence was 39% in this group coinciding with the overall B 19 seroprevalence of 39.9%.

In this study we have found that poor standards of living, low socioeconomic status, poor education levels, overcrowding, poor hosing, and inadequate residential conditions predispose to Parvovirus infection. The association between income and occupation for anti-B19 IgG positivity was also observed Table 2.The unskilled laborers had highest prevalence in low and middle-income groups (48.5% and 58.7%, respectively). This was significantly high (*P*<0.01) in comparison to other occupation groups. It was 33%, 34%, and 38.6% in service, business, and other groups respectively in the low-income group. In the middle-income group, it was 41.8%, 33.3%, 33.2%, and 38.3% in service, skilled personnel, business, and other occupation groups. It was also evident that in low income group persons the prevalence was highest among the illiterates (47.4%) followed by the low educated (36.4%) and the highly educated (28.6%), and the difference was statistically significant (*P*<0.01). Association between occupation and education in terms of B 19 seroprevalence showed that the illiterates and low educated groups maintained a high prevalence (45% and 52.3%, respectively) in unskilled labors and differed significantly (*P*<0.01) from highly educated (37.5%) in the same occupation group [Table 3]. The seropositivity was also assessed according to the number of family members in the house of every donor Table 3. The prevalence of B-19 antibodies was high in donors (54.9%) who were illiterate and were residing in small houses (less than three rooms) as compared to illiterate donors (27.3%) staying in larger houses (more than three rooms) and the difference was statistically significant (*P*<0.01). The prevalence of B19 IgG was higher (60%) in individuals with large family size (> 15 members) (*P*<0.01) in comparison to persons with small family (1 to 5 members) size in which a prevalence of 40% was observed. The overall prevalence did not differ significantly (P>0.05) in persons exposed to animals (38.3%) and who were not exposed (41.3%). Maximum positivity was 46.5% (127 out of 273), observed in winter season, which was significantly higher (*P*<0.01) than the prevalence in rainy season 34.6% (206 of 536) and spring season 38.4% (66 of 191).

**Table 2:** Distribution of anti B19 IgG positivity in relation to income and occupation

Occupation	Income (thousand per month)						
	Low (< 2)		Middle (2-8)		High (> 8)	
	Positive (%)	Total	Positive (%)	Total	Positive (%)	Total
Service	39 (33)	118	72 (41.8)	172	19 (73)	26
Unskilled labourer	49 (48)	101	12 (58.7)	20	00	00
Skilled labourer	51 (45.5)	112	02 (33.3)	05	01 (100)	01
Business	32 (34)	94	51 (33.2)	120	16 (55)	29
Other	62 (38.6)	159	15 (39.5)	39	01 (25)	04
Total	233 (39.8)	584	152 (42.6)	356	37 (61.6)	60

**Table 3:** Distribution of anti B19 IgG positivity by education and size of the house

Education	House size	Positive (%)	Total
Illiterate	Small	39(54.9)	71
	Large	41(27.3)	150
	Total	80(36.1)	221
Low educated	Small	45(47.3)	95
	Large	57(39.3)	145
	Total	102(42.5)	240
Highly educated	Small	97(41.1)	236
	Large	119(39.3)	303
	Total	216(40)	539

## Discussion

Blood is a biologically active material. A spectrum of blood-borne infectious agents is transmitted through transfusion of infected blood donated by apparently healthy and asymptomatic blood donors. The diversity of infectious agents includes hepatitis B virus (HBV), hepatitis C virus (HCV), human immunodeficiency viruses (HIV-1/2), human T-cell lymphotropic viruses (HTLV-I/II), cytomegalovirus (CMV), parvovirus B19, West Nile Virus (WNV), dengue virus, trypanosomiasis, malaria, and variant CJD.[20,21]

The seroprevalence found in this study (39.9%) of North Indian blood donors is lower than the prevalence reported from developed countries such as Europe[12] and England[13] but higher than that of Singapore 16.2%.[16] In most of the countries, B19 virus infection occurs during childhood and by the age of 15 years, approximately 50% of children have anti B19 antibody. B19 prevalence may further increase during lifetime and reach values higher than 85% in elderly. In another study the North African population was reported to have a prevalence of 64% and the Belgian population had a prevalence of 75%.[15] The prevalence in Japan was found to be 55% by ELISA testing using monoclonal antibodies and viral antigen[22] and after outbreaks of erythema infectiosum the rates varied.[23] Though the trend of prevalence did not differ much in India also, the lower prevalence in elderly age group was found. The explanation to this finding could be due to the fact that the maximum age of donors in this study was 60 years and thus the prevalence in still older people was not studied. India being a tropical country with long duration of summer season and with high temperature, it may not be suitable for the transmission of virus during this season. In the colder countries where epidemics of viral infection have been observed it occurred during winter season.[18]

Since the Drugs and Cosmetic act of India does not allow accepting donors less than 18 years and more than 60 years of age for blood donation, the age range was in this limit only. The reason for most donors being young is that in India most of the donations are from close relative as replacement donors and the persons in younger age group being young and in better health take the responsibility and donate blood. The total number of female donors is small in this study due to several reasons. In our hospital most of the patients are referred from distant rural and urban are as for tertiary care and in India males are the ones who accompany the patients and are available for blood donation. Among females who do come forward for blood donation about 70% were rejected, with most common cause for deferral being anemia (23%) followed by low body weight (<45 kg; 7.5%) and lactation or pregnancy (2.6%). The reason for male preponderance to B19 IgG antibodies positivity could also be explained by the fact that in India males are more exposed to occupational hazards as they are the working and earning members of most Indian families. In this study the prevalence was 40% in the males of age group 18-30 years, 38.5% in the age group 30-40 years, 46.03% in age group 40-50 years, and 52% in the age group 50-60 years. In a study from Japan it was demonstrated that the antibody positivity rate was approximately 40% among younger donors (16-20 years), gradually increased among middle-aged men (30-39 yr.) and reached its highest levels (80%) among older donors (>50 years of age).[19] We have found similar results in younger donors but the prevalence in older donors is not so high. This could be probably due to the age limitation (up to 60 years) in our study group. Moreover, India is a tropical country while Japan is a colder country. Increased prevalence has been observed in countries with temperate climate from winter to early summer season. Parvovirus prevalence was not affected by marital status of the donors as was expected, since the sexual route does not spread this infection.

World Health Organization (WHO) proposed a definition of standards of living “Income, occupation, standard of housing, sanitation, nutrition and the level of provision of health, educational, recreational, and other services may all be used individually as measures of socioeconomic status and collectively as an index of the standard of living.” Keeping the above variables in mind the association of the prevalence of anti-B19 IgG antibodies and socioeconomic status of the subjects was established. The higher positivity among manual labors including farmers could be explained by their poor living conditions such as poor housing overcrowding poor income and low education levels. The outdoor activities could lead to more exposure and thus contact with infectious agents and could be a reason for higher prevalence among this occupational group. No previous study has been done to define parvovirus prevalence in terms of socioeconomic status of blood donors; however, considerable work needs to be done in this regard. Education is a major factor influencing health status. The world map of literacy closely coincides with the maps of poverty, malnutrition, ill health, and high mortality rates. Studies have indicated that education, to some extent compensates the effects of poverty on health, irrespective of the availability of health facilities. In this study also illiteracy and low education levels had similar influence on the prevalence rate.

Large family size and small house size were important variables affecting the prevalence of B-19 IgG antibodies. Most of our donors had large family size with more than six members in each family (60.8%). It is not an uncommon occurrence in a populous country like India where joint family systems exist and overcrowding invariably occurs in quite a lot of households. Overcrowding leads to a higher and faster spread of infection through the respiratory route.

Thus, it was found that the prevalence of anti-B19 IgG antibodies is significantly higher in individuals with poor standards of living and low socioeconomic status. To the best of our knowledge, this is the first study of this kind in India. Since mainly respiratory droplets spread parvovirus B19, similar trends have been documented in the epidemiology of communicable diseases spread through respiratory route such as diphtheria, rubella, influenza, pertusis, meningococcal meningitis, and tuberculosis. The risk of exposure is greater in lower social classes, living in overcrowded and poor housing conditions. The lack of education, large families, and poor qualities of life are the predisposing factors for such infections. Blood-borne infections like hepatitis B are also influenced by the socioeconomic status. The prevalence is lowest in areas of high standards of living (Australia, Europe) and highest in countries of areas with poor socioeconomic status and living conditions (China, South East Asia). Epidemiological studies have shown that health and disease is not equally distributed in social classes. Individuals in upper social classes have longer life expectancy and better health than those in lower social classes, but certain diseases such as diabetes, coronary heart disease, and hypertension are more common in the former group. Social class difference has also been observed in utilization of health care services; however, social classification varies from country to country.

In this study, the observation of a significantly higher prevalence in the winter season could be related to the preferential occurrence of epidemics during winter or spring in temperate climates and points to a more effective air-borne transmission of virus at colder temperatures.[13] Since the span of this study was 9 months, the epidemiology of seasonal variation in summer months could not be studied.

The incidences of viraemic blood donors prior to PCR-based assays were reported to be very low, about 1 in 30,000 to 50,000. Now with advancements in PCR assays and especially nested-PCR, the incidence of B19 viraemia has been found in 0.03% to 0.6% and even higher percentage of blood donors.[24,25] This frequency is sufficient to contaminate plasma pools used in preparation of factor VIII or IX concentrates. Thus multi-transfused hemophiliacs receiving factor concentrates are likely to be greatest victim of transfusion-transmitted B19 infection. Lastly, it should be borne in mind that seropositive donors are immune and the donor unit contains neutralizing antibodies.

More than 25 years after the discovery of the parvovirus B19, the issue of the safety of blood components and the screening of this virus in blood donations is still debated. However, the existence of persistently B19-infected individuals carrying B19 DNA despite the presence of specific IgG (estimated at 1% of blood donors) could constitute a potential threat for transfused immunocompromised recipients.[26] The screening of such donors, which could be performed through a very highly sensitive NAT (nucleic acid amplification test), would be justified only if the infectivity of such blood donations is demonstrated. If not, a screening of blood donors positive for B19 IgG would be a sufficient preventive measure.[27]
